# Historical reconstruction of the earliest enterovirus A71 epidemics in Japan in the 1960s

**DOI:** 10.1017/S0950268826101435

**Published:** 2026-04-20

**Authors:** Xinhua Chen, Nathanaël Hozé, Margarita Pons-Salort

**Affiliations:** 1 MRC Centre for Global Infectious Disease Analysis, School of Public Health, Imperial College London, London, United Kingdom; 2 Université Paris Cité and Université Sorbonne Paris Nord, Inserm, IAME, F-75018 Paris, France

**Keywords:** emergence, enterovirus A71, hand, foot and mouth disease, mathematical model, serology, force of infection

## Abstract

Enterovirus A71 was first isolated in California in 1969, with the earliest retrospective detection traced back to 1963 in the Netherlands, but its early spread remains unclear. Using age-specific seroprevalence data from children aged 1–10 years in Kawasaki City, Japan, collected annually from 1966–1973, we applied serocatalytic models to estimate annual force of infection during 1959–1973. Several models were tested, incorporating different assumptions about time-varying force of infection, age-dependent susceptibility, and seroreversion, to identify the best fit to the data. Model comparison identified the models with independent annual infection probability or two distinct outbreak periods, both including age-dependent force of infection and seroreversion, as optimal. All top models consistently identified two major transmission periods: 1961–1962 and 1968–1969. The two-outbreak model estimated mean attack rates of 21.8% and 37.8% for the earlier and later outbreaks under seroreversion, and 19.8% and 34.9% under age-dependent force of infection. Our findings provide evidence of enterovirus A71 circulation in Japan during two distinct periods in the 1960s, coinciding with early detections in Europe and the USA, suggesting global distribution by that decade. This study underscores the value of testing archived sera for reconstructing pathogen emergence and spread.

## Introduction

Enterovirus A71 (EV-A71) is a single-stranded, positive-sense RNA virus belonging to the enterovirus genus in the family Picornaviridae [1]. As one of the main pathogens causing hand, foot, and mouth disease (HFMD) in children, EV-A71 infections can cause mucocutaneous and respiratory manifestations, such as fever, sore throat, and multiple rashes on palms, soles, buttocks, and oral cavity. In rare instances, EV-A71 infection can result in more severe neurological and systemic manifestations, like aseptic meningitis, acute flaccid paralysis, and encephalitis [2].

**Figure 1.**
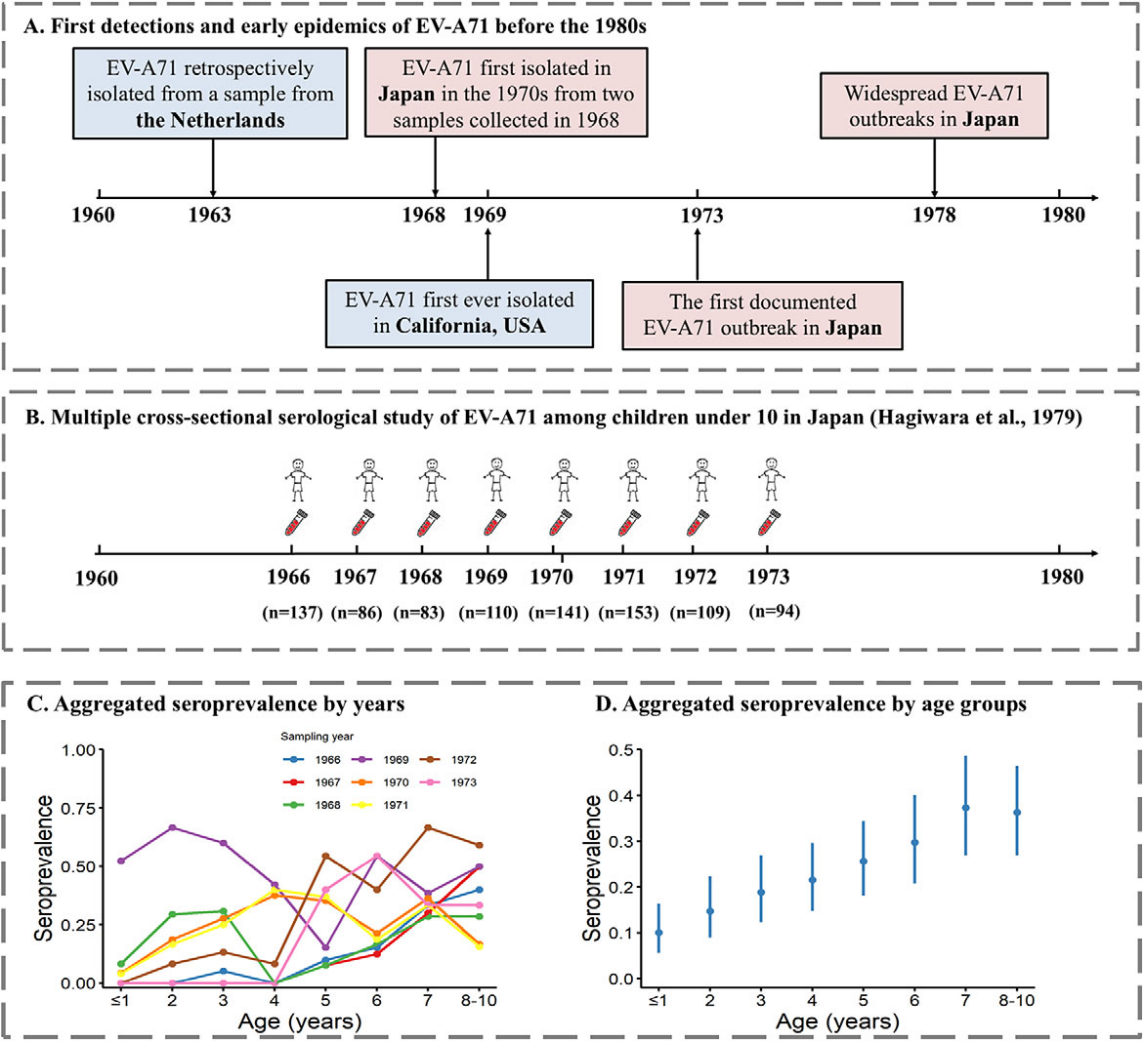
Timeline of early EV-A71 detections worldwide and serology study in Japan. (a) Early detections and epidemics of EV-A71 worldwide and in Japan. (b) Sampling of EV-A71 serology study in Japan by Hagiwara et al. [3] (c) Seroprevalence by age for different sampling years from Hagiwara et al. [3]. (d) Age-specific seroprevalence aggregated across all sampling years.

EV-A71 was first recognized as a new enterovirus serotype when isolated from more than 20 patients with disease of the central nervous system (CNS), mostly encephalitis and meningitis, in California, between 1969 and 1972 (Figure 1a) [4]. However, an extensive study of the epidemiology of EV-A71 in the Netherlands, which retrospectively tested isolates from 1963 onwards, identified EV-A71 in one isolate from 1963 (Figure 1a) and in a few others from 1965, 1966, and 1967 [5]. In the 1970s, outbreaks of diseases associated with EV-A71 of different sizes and severity occurred around the globe, including in Australia [6], the USA [7], Sweden [8], Bulgaria [9, 10], and Hungary [11]. Most cases of these outbreaks reported neurological symptoms, often similar to poliomyelitis, although a few cases of skin rashes were also described [6, 8]. In Japan, two large-scale outbreaks of HFMD associated with EV-A71 occurred in 1973 and 1978, with most patients experiencing typical symptoms of HFMD (including skin manifestations) and a low incidence of disorders of the CNS (even lower in 1978 than in 1973), compared to previous and concurrent EV-A71 outbreaks in Europe and elsewhere [12, 13]. These were the first large-scale outbreaks of HFMD associated with EV-A71 ever reported [12, 13]. In the 1980s and early 1990s, sporadic epidemics of EV-A71 continued to occur (e.g. in Hong Kong, China, in 1985, and Australia in 1986) [14, 15]. In 1997, over 2500 HFMD cases and 34 deaths were reported in Sarawak, Malaysia [16], which marked the beginning of a series of large-scale outbreaks of HFMD in Southeast Asia [17]. According to data from China’s national enhanced surveillance system, between 2008 and 2012, there were approximately two million HFMD cases each year, resulting in around 500–900 deaths annually [18].

Although a few species A enteroviruses can cause HFMD (including EV-A71, Coxsackievirus (CV)A6, CVA10, and CVA16), EV-A71 infections are responsible for the majority of severe and fatal cases [18], and EV-A71 is now considered the most neurotropic enterovirus after the three poliovirus serotypes. In 2015 and 2016, China approved the use of three inactivated EV-A71 vaccines, which had shown high efficacy against EV-A71–associated HFMD during clinical trials [19, 20]. However, vaccine coverage is low, and questions about the target population, optimal immunization schedule, and long-term effectiveness continue to exist. Today, EV-A71 infections remain a public health concern, posing a significant disease burden, especially in the paediatric population in Southeast Asia.

In order to clarify whether EV-A71 circulated in Japan before the first large epidemic of 1973, a retrospective serosurvey of neutralizing antibodies against EV-A71 among healthy children was conducted in the Takatsu area, Kawasaki City (near Tokyo) [3]. Data from this serosurvey, with detailed age stratifications among children under 10 years old (yo) and annual sampling between 1966 and 1973, provide us with an excellent opportunity to reconstruct the historical circulation of EV-A71 in this area. Here, we use serocatalytic models and the seroprevalence data reported in Hagiwara et al. [3] to reconstruct the historical circulation of EV-A71 in Kawasaki City, Japan, and quantitatively assess the likelihood of historical EV-A71 outbreaks during the 1960s.

## Methods

### Data

Age-stratified seroprevalence data were extracted from a retrospective cross-sectional serology study in Japan that tested archived serum samples collected each March between 1966 and 1973 [3]. This study recruited 913 healthy children aged 0–10 years in the Takatsu area of Kawasaki City, near Tokyo, with an average of 114 (range: 83–141) participants recruited each year **(** Figure 1b **)**. A live virus neutralization assay was used to detect EV-A71–specific antibody titres [21]. Briefly, serial two-fold dilutions of serum were prepared in duplicate, starting from 1:4 to 1:512. Serum was mixed with titred virus (BrCr strain) in equal volume and then incubated at 36°C for 3 h and placed overnight at 4°C. After incubation, a continuous cynomolgus monkey kidney cell line (CMK1-S1) was added, and the resulting mixture was incubated at 36°C. The results were read on the fifth and seventh day after inoculation, and 50% endpoints were calculated. The seropositivity cut-off was set as 1:4.

The number of samples tested per age class and year is available in Hagiwara et al. [3], and the aggregated age-specific seroprevalence per age class and year is displayed in figures [3]. We used the online data extractor WebPlotDigitizer, version 4.8, to extract the crude seroprevalence for each age group and year, and then derived the number of seropositive samples by multiplying the extracted seroprevalence by the number of samples tested [22]. Age-specific seroprevalence was provided aggregated for 2 consecutive years, 1966–1967 and 1970–1971, because age-specific seroprevalence patterns were very similar, according to the authors [3]. For those years, we proportionally estimated the sample size for each age group based on the total number of samples tested each year.

### Serocatalytic models

Serocatalytic models are designed to estimate the force of infection (FOI), which represents the per-capita rate at which susceptible individuals become infected, based on age-stratified seroprevalence data [23]. Here, we used the ‘RSero’ package within R (version 4.4.1) to test different models that make different assumptions about how the FOI changes over time and/or age [24]. We considered three types of models: time-independent model, one-outbreak model, and two-outbreak model. Specifically, the time-independent model assumes that the FOI changes every year (hereinafter referred to as ‘independent model’), and a separate posterior distribution is estimated for the FOI in each year, resulting in a relatively large number of parameters in our setting. Because the FOI is reconstructed retrospectively from the sampling period, earlier years are informed by progressively fewer observations, reducing parameter identifiability and increasing posterior uncertainty. The outbreak model assumes that there have been a fixed number 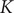 of epidemics in the past, where 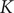 is a positive integer. When 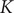 = 1, it is referred to as the ‘one-outbreak model’, and when 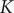 = 2, it is referred to as the ‘two-outbreak model’. Given a fixed value of 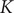 , posterior inference is performed on a set of two-outbreak-specific parameters, including outbreak intensity ( 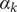 ) and timing ( 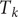 ). This parsimonious parameterization reduces model complexity relative to the independent model, leading to lower parameter uncertainty and a reduced risk of overfitting. For each of the three time-varying FOI models, we further tested four versions of the model: 1) the basic model, 2) a model with age-dependent FOI, 3) a model with seroreversion, and 4) a model with both age-dependent FOI and seroreversion. Therefore, a total of 12 models were considered. A summary of all models and their assumptions is available in Supplementary Tables S1 and S2.

### Model fitting, selection and comparison

All models were fitted to the seroprevalence data using Markov Chain Monte Carlo (MCMC), with the No-U-Turn sampler (NUTS) sampling algorithm to update the parameters. Four independent chains with 5000 iterations were used, with the first 2500 corresponding to the warm-up period. Convergence was evaluated both visually by inspecting the MCMC chains, and through the Rhat values and effective sample size (ESS) of the parameters. Specifically, the Rhat values should be below 1.01, while the bulk and tail ESS should exceed 400 for each parameter. Leave-One-Out Cross-Validation (LOO) and Deviance Information Criterion (DIC) were used for model selection [25]. To estimate the credible intervals (CrIs) for the parameters, we performed posterior sampling by drawing 1000 samples from the posterior distribution and extracted the 2.5th and 97.5th percentiles of these samples.

## Results

### Aggregated seroprevalence

Age-specific seroprevalence for each sampling year showed generally higher estimates in older children than in younger children. However, in contrast to other years, in 1969, seroprevalence in children under 3 yo exceeded 50% (Figure 1c). Across all years, the overall seroprevalence increased with age, from 10.1% (95% confidence interval (CI): 5.6%–16.3%) for children aged 0–1 years to 36.3% (95% CI: 27.0%–46.4%) for children aged 8–10 years (Figure 1d).

### Model selection

We fitted a total of 12 serocatalytic models to the data, and based on the LOO and DIC criterion, we found that the independent model with age-dependent FOI was the best model, closely followed by the independent model with seroreversion and the independent model with both age-dependent FOI and seroreversion (Supplementary Table S3). The small differences in LOO and DIC values among these top three models suggest that they are statistically indistinguishable in terms of predictive performance. The two-outbreak model with age-dependent FOI and the two-outbreak model with seroreversion ranked fourth and fifth, respectively. The 4 one-outbreak models ranked lowest in both LOO and DIC comparisons, clearly indicating poor performance relative to the other models. Given these results, we decided to present and discuss the results of the independent and two-outbreak models with either age-dependent FOI (Models 2 and 10) or seroreversion (Models 3 and 11).

### FOI estimates

**Figure 2.**
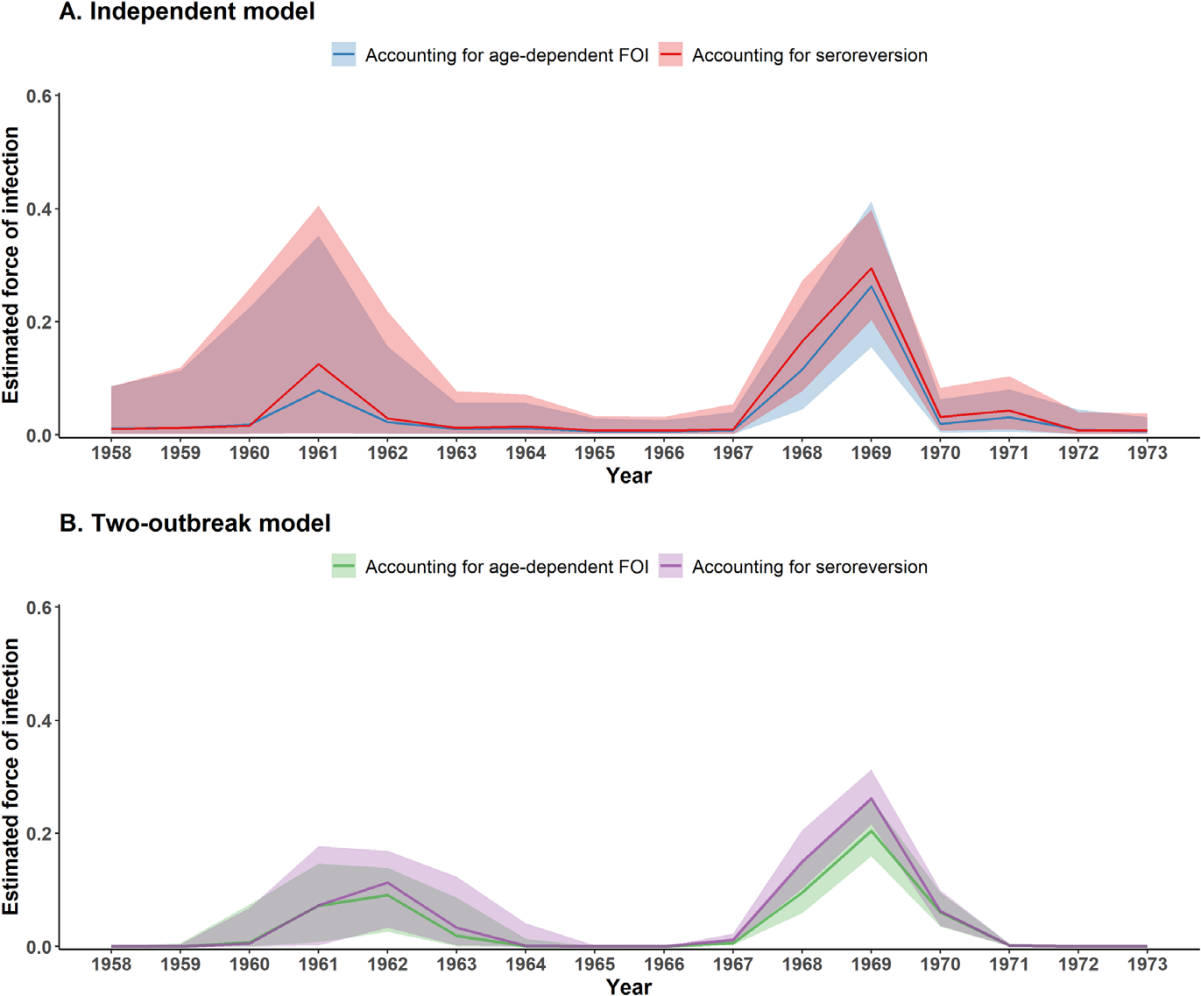
Estimated FOI between 1958 and 1973 obtained with serocatalytic models. (a) Independent model. (b) Two-outbreak model. Coloured lines and envelopes indicate the annual FOI estimated median and corresponding 95% CrIs.

Similar temporal trends in FOI estimates were observed across the independent and two-outbreak models (Figure 2). All four models identified two main periods of EV-A71 circulation, one around 1968–1969 and an earlier one around 1961–1962. However, the annual FOI estimates from the two-outbreak model showed less uncertainty than those from the independent model, likely due to its simpler parameterization and the imposition of a strong temporal structure. As a result, the independent model resulted in larger uncertainty in the FOI estimates of the earliest outbreak. This uncertainty is evident in the wide variability of individual FOI trajectories (Supplementary Figure S1). Across the two-outbreak models, individual FOI trajectories showed a highly consistent timing for the 1968–1969 outbreak and a bit more variability in the timing of the earliest outbreak. In contrast, the individual FOI trajectories estimated with the two independent models showed greater variability in the timing of the 1968–1969 outbreak, which continued to increase towards the earliest outbreak, with the timing and magnitude of the FOI estimates varying considerably across trajectories.

The models with an age-dependent risk of infection estimated that the FOI increased with age, with a parameter for the exponential increase of 0.01 (95% CI: −0.05–0.08) for the independent model and 0.04 (95% CI: 0–0.09) for the two-outbreak model. These correspond to a 1.15-fold (95% CI: 0.66–1.86) increase in FOI at age 10 compared to age 1 for the independent model and a 1.43-fold (95% CI: 1.02–1.96) increase for the two-outbreak model (Supplementary Figures S2 and S3). The CrI for the independent model included 1, indicating little to no evidence for age-related differences in risk. In contrast, the two-outbreak model excluded 1 from its CrI, suggesting a modest but statistically supported increase in infection risk with age. The models with seroreversion estimated similar seroreversion rates of 0.03 (95% CrI: 0.01–0.08) for the independent model and 0.02 (95% CrI: 0.01–0.06) for the two-outbreak model (Supplementary Table S4). These correspond to 13.9% (95% CrI: 4.8%–33.0%) and 9.5% (95% CrI: 4.8%–25.9%) of children losing detectable antibodies within 5 years, respectively, for the independent and two-outbreak models. Trace plots and posterior density plots for all estimated parameters are shown in Supplementary Figures S4–S11.

The outbreak models are parameterised to return the overall probability of infection over the course of an outbreak, also referred to as the attack rate. In the two-outbreak model with seroreversion, the estimated attack rates were 21.7% (95% CrI: 15.9%–29.2%) for the earlier outbreak and 38.7% (95% CrI: 34.1%–43.9%) for the later outbreak (Figure 2b). For the two-outbreak model with age-dependent FOI, the corresponding attack rates were lower, 19.8% (95% CrI: 0.1%–27.1%) and 34.9% (95% CrI: 27.5%–44.0%) for the earlier (1960–1964) and later outbreaks (1967–1971), respectively (Figure 2b **)**. To compare these estimates with those from the independent model, we computed the overall probability of infection over the period 1967–1971 (i.e. the years with a non-zero FOI) using the annual FOI estimates from the independent model (Figure 2a). The overall probability of infection was higher with the independent model compared to the two-outbreak model: 41.9% (95% CrI: 36.4%–47.9%) for the seroreversion model and 36.9% (95% CrI: 24.8%–50.3%) for the age-dependent model. We did not compute the attack rate for the earlier outbreak using the independent model, due to substantial uncertainty in FOI estimates prior to 1965.

### Modelled seroprevalence

**Figure 3.**
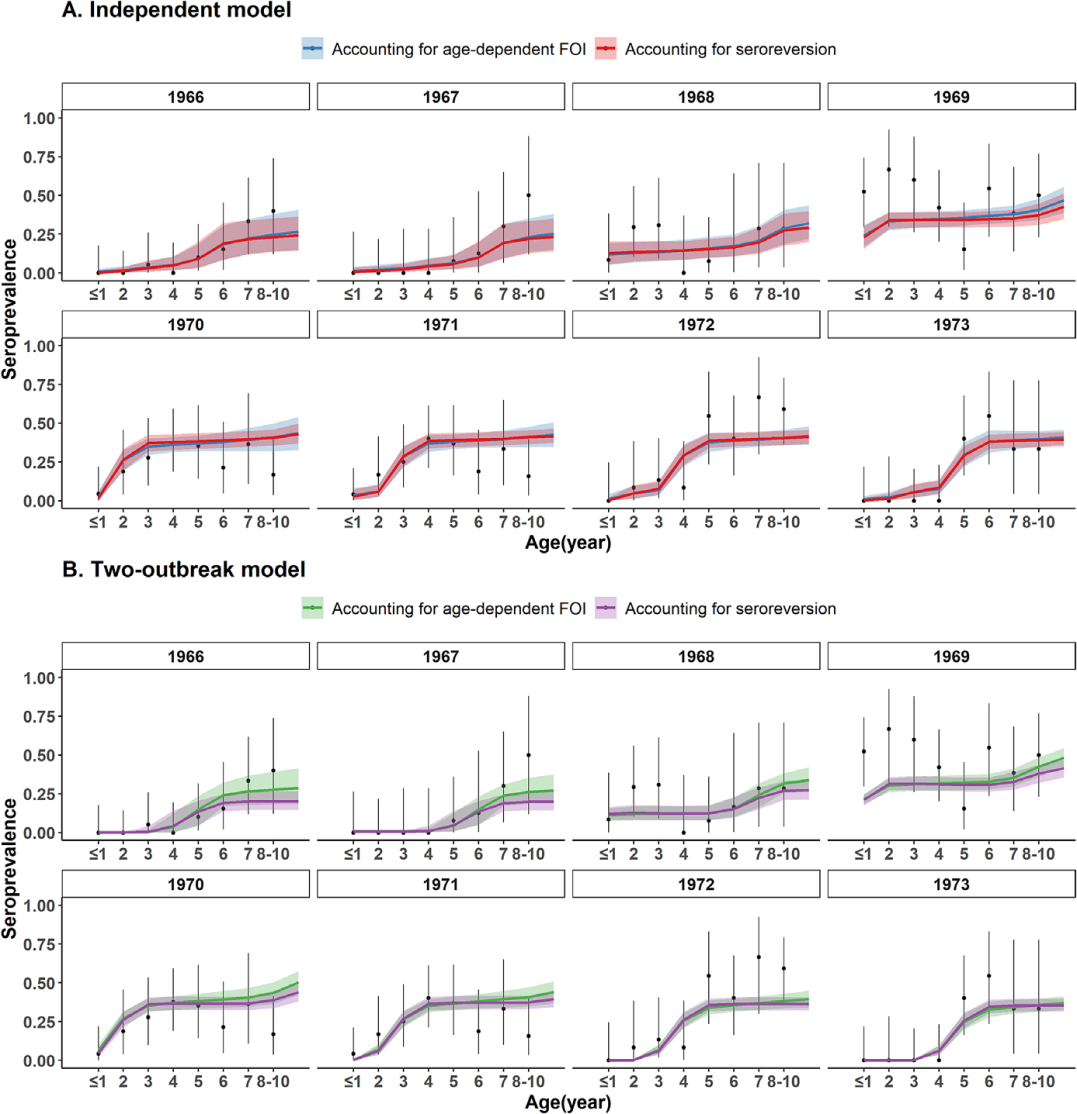
Model fit to EV-A71 seroprevalence data. (a) Independent model. (b) Two-outbreak model. Black points indicate the original seroprevalence data from Hagiwara et al. and intervals are the 95% binomial CIs. Coloured lines and envelopes are the mean values and 95% CrIs estimated with the serocatalytic models.

All four models explained the age-patterns of seroprevalence well and provided very similar fits to the data (Figure 3). Seroprevalence was estimated close to zero in the 1–3 yo in 1966 and in the 1–4 yo in 1967, and then increased with age, reaching 26.5% (95% CrI: 15.1%–40.9%) in the 8–10 yo with the independent model with age-dependent FOI and 24.3% (95% CrI: 14.5%–36.5%) with the independent model with seroreversion. With the two-outbreak model, seroprevalence increased from less than 1% in children aged 1–3 years to 27.3% (95% CrI: 18.7%–37.5%) and 19.8% (95% CrI: 14.4%–26.4%) in those aged 8–10 years in 1967, respectively, when accounting for age-dependent FOI and seroreversion. Seroprevalence then increased in the younger age classes when it almost plateaued at around 12% in 1968 and 31% in 1969 for 2 yo children. In 1970 and 1971, seroprevalence increased sharply with age for children under 4 yo, and then plateaued between the ages of 4 and 10 years at around 40%, in agreement with the outbreak around 1968–1969. A similar age pattern, shifted in age, was estimated for 1972 and 1973, when seroprevalence in the 1 yo and in the 1–2 yo, respectively, was estimated null or close to zero.

### Seroconversion across age

**Figure 4.**
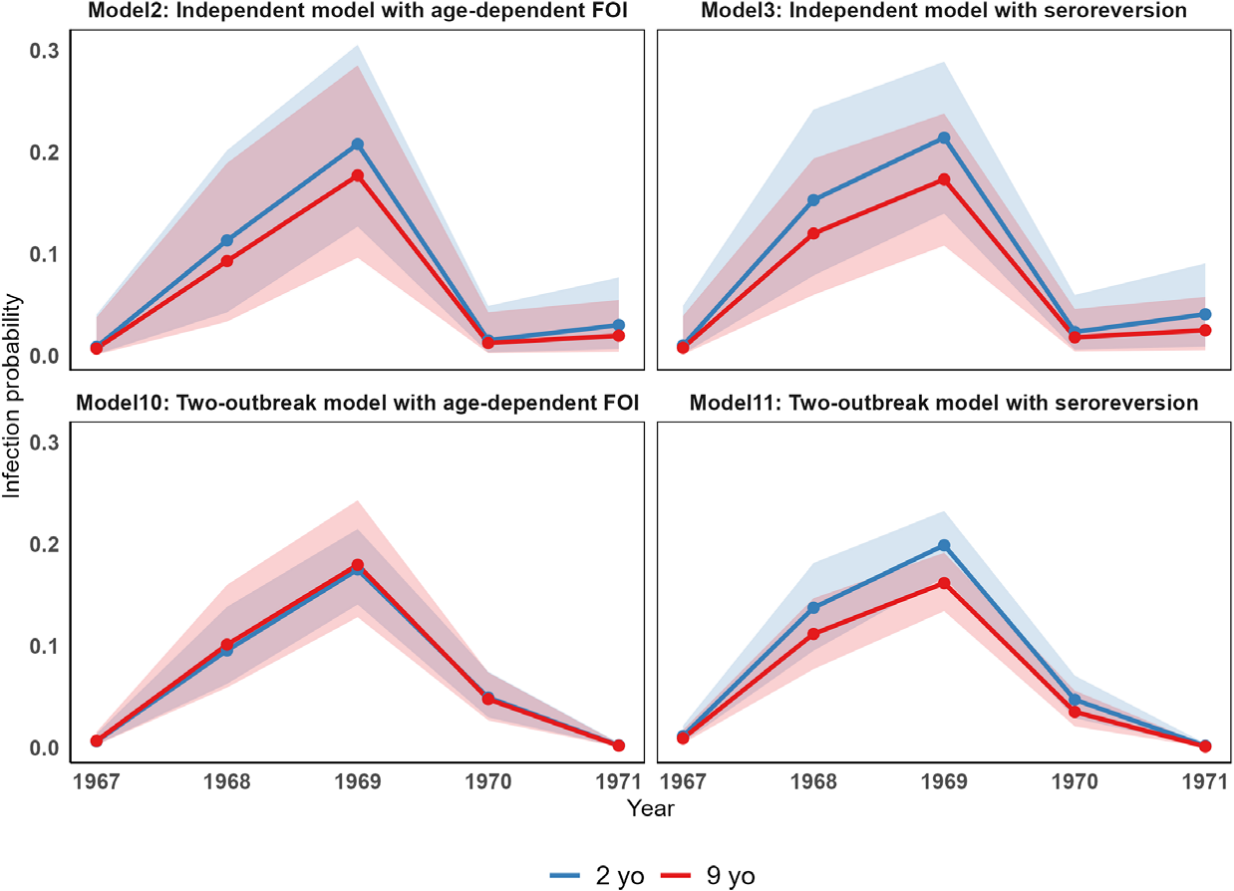
Differences in the risk of seroconversion through age across the independent and two-outbreak models.

Although the two versions of the two models (independent and two-outbreak) provided similar results in terms of the annual FOI estimates and model fits, the two underlying assumptions (age dependency in the risk of seroconversion vs. seroreversion) may result in different dynamics of seroconversion across different age groups. To investigate these differences through age under the four models, we computed the probability of infection for a typical 2-yo individual and a typical 9-yo individual (Figure 4). The two models with seroreversion estimated a higher probability of seroconversion for the 2 yo than the 9 yo. This pattern aligns with a smaller proportion of 9 yo being susceptible, a constant risk of infection across age, and a low seroreversion rate. In contrast, the two models with age-dependent FOI showed smaller differences in the probability of infection between the two age groups. Notably, in the two-outbreak model, the probability was almost identical for both age classes. This pattern may be explained by the estimated higher risk of seroconversion with age (which is higher for the two-outbreak model than that for the independent model) that counterbalances the decreasing proportion of susceptible with age and the absence of seroreversion.

## Discussion

Using serocatalytic models and publicly available serological data, this study estimates the timing and magnitude of the earliest EV-A71 outbreaks ever reported in Japan. We estimated that during the decade of the 1960s, there were two main circulation periods in Kawasaki City (near Tokyo), one around 1961–1962 and the other around 1968–1969. The timing of these outbreaks coincides with the first detections of EV-A71 reported from clinical samples around the globe: in 1963 from one sample in the Netherlands [5], followed by a few other detections between 1965 and 1967; in 1967 from six cases of aseptic meningitis in Sweden [8]; and in 1969 from a patient with encephalitis in California, USA [4], followed by other isolations from patients with CNS disease during 1970–1972.

Bayesian evolutionary analysis of the VP1 capsid sequence estimated that EV-A71 diverged from its closely related ancestor CVA16 around 1940, with relatively large uncertainty around that estimate (95% CI: 1928.8–1952.2) [26]. EV-A71 is therefore a relatively recent human pathogen. However, the exact location of emergence and initial routes of spread remain uncertain. Our results, jointly with the currently available literature, suggest that by the second half of the 1960s, EV-A71 had already widespread through Europe, the USA, and Japan.

Our results are robust to the model structure. Both the time-independent and two-outbreak models (either with age-dependent FOI or seroreversion) provided highly consistent estimates of the FOI between 1964 and 1973. There was a bit more uncertainty around the FOI estimates between 1959 and 1963, when less data informed the parameters. Nevertheless, both models clearly supported the circulation of EV-A71 during two different periods between 1959 and 1973. In addition, with seroprevalence >10% in those aged 5 years and older in the first time-point of Hagiwara et al. in 1966, it is likely that EV-A71 already circulated before 1966 in Japan.

A countrywide HFMD epidemic associated with CVA16 occurred in Japan between mid-1969 and the end of 1970 [27]. However, it was reported that a substantial proportion of all tested specimens was not positive for CVA16 [27]. In addition, isolates from those years were not neutralized by antiserum against the CVA16 prototype. It may be that EV-A71 (which was not recognized as a new enterovirus serotype by that time) co-circulated with CVA16 during the time of that outbreak years. In addition, EV-A71 circulation is also corroborated by the first isolation of this virus from two samples collected in 1968 [3]. Japan also experienced a nationwide EV-A71 outbreak in 1973 [28]. However, our study failed to capture this outbreak, probably because our extracted data for 1973 were based on samples from March and the outbreak occurred later in the year [28]. There were no seropositive children younger than 4 years in 1973 in our dataset.

Interestingly, our findings are consistent with EV-A71 case-based surveillance data from Japan from 2000 onwards, as well as estimates from two modelling studies that fitted transmission models to those data [29, 30]. These studies reported recurrent EV-A71 outbreak cycles occurring approximately every 4–5 years, with major outbreaks infecting around 20%–30% of the population. Similarly, our two-outbreak model estimated a mean attack rate of 21.8%–37.8%, alongside an approximately 5-year interval between the two reconstructed outbreaks. While the previous studies considered the entire population, our analysis focused on children under 10 years of age; however, given that EV-A71 infections predominantly occur in young children, the results remain broadly comparable. Together, these comparisons suggest that the reconstructed epidemiological patterns for EV-A71 infection in Kawasaki City in the 1960s are consistent with those observed in Japan over the last two decades.

In our study, the independent and the two-outbreak models with seroreversion performed better than their counterparts without it. Specifically, we estimated a seroreversion rate of 0.03 (95% CrI: 0.01–0.08) and 0.02 (95% CrI: 0.01–0.06) for the independent and the two-outbreak models, respectively. This corresponds to a mean duration of antibody detection of 32.4 (95% CrI: 13.1–95.4) and 42.8 (95% CrI: 18.6–123.4) years, respectively. However, the decay of seropositivity may not be very well estimated, given that we only have data for individuals up to 10 years of age. Furthermore, the long duration of seropositivity could also partly be explained by the low seropositivity cut-off (1:4) used in the serological study. Additionally, the long EV-A71 duration of seropositivity is supported by a study that reported that up to 97% of young children maintained neutralizing antibody titres above 1:8 even 5 years after receiving a single dose of the inactivated EV-A71 vaccine [31]. Moreover, findings from a seroepidemiology study conducted in Southern China between 2013 and 2018 reported that following natural infection, geometric mean titers (GMTs) among seropositive individuals slowly declined with age [32]. However, GMTs remained above 64 in children aged 12 years, indicating long-lasting EV-A71 antibody titres and possibly low seroreversion rates. Given the wide CrIs around our estimated seroreversion rates, as well as data limitations noted above, it is difficult to draw definitive conclusions about the true underlying seroreversion rate.

Our study has several limitations, mainly inherent to the data used to inform the models. Firstly, data were extracted from a published study that reported data twice for 2 consecutive years together. However, the authors explained in their paper that the age profile was very similar for those 2 years, and that was the reason why they plotted it together. This may slightly affect our estimates of the timing of inferred outbreaks. Second, we do not have detailed information on the children who were sampled; only their age and that they were healthy were available, but we do not know to what extent they were representative of the children who lived in Kawasaki City at the time of data collection (1966–1973).

In conclusion, our study provides evidence of the first EV-A71 outbreaks in Japan, by reconstructing historical circulation from detailed retrospective seroprevalence data from consecutive cross-sectional surveys that sampled children over 8 consecutive years. This captured well the first infections and allowed reconstructing EV-A71 circulation on a fine timescale. This work highlights the value of testing archived serum samples to clarify the emergence and spread of human pathogens.

## Supporting information

10.1017/S0950268826101435.sm001 Chen et al. supplementary material Chen et al. supplementary material

## Data Availability

The data and code are available at https://github.com/cxhhhh24/ev71_foi.git

## Supplementary material

The supplementary material for this article can be found at http://doi.org/10.1017/S0950268826101435.

## References

[1] Wong SSY, et al. (2010) Human enterovirus 71 and hand, foot and mouth disease. Epidemiology and Infection 138(8), 1071–1089. 10.1017/S0950268809991555. 20056019

[2] Ooi MH, et al. (2010) Clinical features, diagnosis, and management of enterovirus 71. The Lancet Neurology 9(11), 1097–1105. 10.1016/S1474-4422(10)70209-X. 20965438

[3] Hagiwara A, et al. (1979) Seroepidemiology of enterovirus 71 among healthy children near Tokyo. Microbiology and Immunology 23(2), 121–124. 10.1111/j.1348-0421.1979.tb00448.x. 223018

[4] Schmidt NJ, et al. (1974) An apparently new enterovirus isolated from patients with disease of the central nervous system. The Journal of Infectious Diseases 129(3), 304–309. 10.1093/infdis/129.3.304. 4361245

[5] Van Der Sanden S, et al. (2009) Epidemiology of enterovirus 71 in the Netherlands, 1963 to 2008. Journal of Clinical Microbiology 47(9), 2826–2833. 10.1128/jcm.00507-09. 19625480 PMC2738086

[6] Kennett ML, et al. (1974) Enterovirus type 71 infection in Melbourne. Bulletin of the World Health Organization 51(6), 609–615. 4377551 PMC2366271

[7] Deibel R, et al. (1979) Central nervous system infections. Etiologic and epidemiologic observations in New York state, 1976–1977. New York State Journal of Medicine 79(5), 689–695. 286166

[8] Blomberg J, et al. (1974) New enterovirus type associated with epidemic of aseptic meningitis and/or hand, foot, and mouth disease. The Lancet 304(7872), 112. 10.1016/S0140-6736(74)91684-5. 4136956

[9] Chumakov M, et al. (1979) Enterovirus 71 isolated from cases of epidemic poliomyelitis-like disease in Bulgaria. Archives of Virology 60(3–4), 329–340. 10.1007/bf01317504. 228639

[10] Shindarov LM, et al. (1979) Epidemiological, clinical, and pathomorphological characteristics of epidemic poliomyelitis-like disease caused by enterovirus 71. Journal of Hygiene, Epidemiology, Microbiology, and Immunology 23(3), 284–295. 231067

[11] Nagy G, et al. (1982) Virological diagnosis of enterovirus type 71 infections: Experiences gained during an epidemic of acute CNS diseases in Hungary in 1978. Archives of Virology 71(3), 217–227. 10.1007/bf01314873. 6285858

[12] Ishimaru Y, et al. (1980) Outbreaks of hand, foot, and mouth disease by enterovirus 71. High incidence of complication disorders of central nervous system. Archives of Disease in Childhood 55(8), 583–588. 10.1136/adc.55.8.583. 6254449 PMC1627055

[13] Tagaya I, et al. (1981) A large-scale epidemic of hand, foot and mouth disease associated with enterovirus 71 infection in Japan in 1978. Japanese Journal of Medical Science and Biology 34(3), 191–196. 10.7883/yoken1952.34.191. 6273621

[14] Gilbert GL, et al. (1988) Outbreak of enterovirus 71 infection in Victoria, Australia, with a high incidence of neurologic involvement. The Pediatric Infectious Disease Journal 7(7), 484–488. 10.1097/00006454-198807000-00007. 2841639

[15] Samuda GM, et al. (1987) Monoplegia caused by enterovirus 71: An outbreak in Hong Kong. The Pediatric Infectious Disease Journal 6(2), 206–208. 10.1097/00006454-198702000-00013. 3562141

[16] Cardosa MJ, et al. (1999) Isolation of subgenus B adenovirus during a fatal outbreak of enterovirus 71-associated hand, foot, and mouth disease in Sibu, Sarawak. The Lancet 354(9183), 987–991. 10.1016/s0140-6736(98)11032-2. 10501361

[17] Solomon T, et al. (2010) Virology, epidemiology, pathogenesis, and control of enterovirus 71. The Lancet Infectious Diseases 10(11), 778–790. 10.1016/s1473-3099(10)70194-8. 20961813

[18] Xing W, et al. (2014) Hand, foot, and mouth disease in China, 2008-12: An epidemiological study. The Lancet Infectious Diseases 14(4), 308–318. 10.1016/s1473-3099(13)70342-6. 24485991 PMC4035015

[19] Adminstration NMP (2015) The Food and Drug Administration approved the production and marketing of enterovirus 71 inactivated vaccine. https://www.nmpa.gov.cn/yaopin/ypjgdt/20151203164201285.html (accessed 26 October 2024).

[20] Li R, et al. (2014) An inactivated enterovirus 71 vaccine in healthy children. The New England Journal of Medicine 370(9), 829–837. 10.1056/NEJMoa1303224. 24571755

[21] Moritsugu Y, et al. (1972) A comparison of micromethod and conventional tube method in detection of polio-neutralizing antibody. Japanese Journal of Medical Science and Biology 25(1), 43–46. 10.7883/yoken1952.25.43. 4338980

[22] Rohatgi A (2024) WebPlotDigitizer 4.8. https://github.com/automeris-io/WebPlotDigitizer (accessed 29 October 2024).

[23] Rees EM, et al. (2022) Estimating the duration of antibody positivity and likely time of Leptospira infection using data from a cross-sectional serological study in Fiji. PLoS Neglected Tropical Diseases 16(6), e0010506. 10.1371/journal.pntd.0010506. 35696427 PMC9232128

[24] Hozé N, et al. (2025) RSero: A user-friendly R package to reconstruct pathogen circulation history from seroprevalence studies. PLoS Computational Biology 21(2), e1012777. 10.1371/journal.pcbi.1012777. 39899643 PMC11809794

[25] Allen DM (1974) The relationship between variable selection and data Agumentation and a method for prediction. Technometrics 16(1), 125–127. 10.2307/1267500.

[26] Tee KK, et al. (2010) Evolutionary genetics of human enterovirus 71: Origin, population dynamics, natural selection, and seasonal periodicity of the VP1 gene. Journal of Virology 84(7), 3339–3350. 10.1128/jvi.01019-09. 20089660 PMC2838098

[27] Tagaya I, et al. (1973) Epidemic of hand, foot and mouth disease in Japan. Japanese Journal of Medical Science & Biology 26(3), 143–147. 10.7883/yoken1952.26.143. 4543070

[28] Tagaya I, et al. (1975) Epidemic of hand, foot and mouth disease in Japan, 1972-1973: Difference in epidemiologic and virologic features from the previous one. Japanese Journal of Medical Science and Biology 28(4), 231–234. 10.7883/yoken1952.28.231. 175202

[29] Pons-Salort M, et al. (2018) Serotype-specific immunity explains the incidence of diseases caused by human enteroviruses. Science 361(6404), 800–803. 10.1126/science.aat6777. 30139872 PMC6559928

[30] Yan X, et al. (2026) Impact of COVID-19 on the transmission dynamics of HFMD associated enterovirus serotypes in Japan: A modelling study of surveillance data. Epidemics 54, 100899. 10.1016/j.epidem.2026.100899. 41734482

[31] Hu Y, et al. (2018) Five-year immunity persistence following immunization with inactivated enterovirus 71 type (EV71) vaccine in healthy children: A further observation. Human Vaccines & Immunotherapeutics 14(6), 1517–1523. 10.1080/21645515.2018.1442997. 29482422 PMC6037439

[32] Yang J, et al. (2022) Seroepidemiology of enterovirus A71 infection in prospective cohort studies of children in southern China, 2013-2018. Nature Communication 13(1), 7280. 10.1038/s41467-022-34992-1. PMC9701185 36435844

