# Supplementary material for: Historical reconstruction of the earliest enterovirus A71 epidemics in Japan in the 1960s
Source: Epidemiol Infect. 2026 Apr 20;154:e57. doi: 10.1017/S0950268826101435 (PMC13161800; doi:10.1017/S0950268826101435)
Supplement: Chen et al. supplementary material [file S0950268826101435sup001.docx]

**Historical reconstruction of the earliest enterovirus A71 epidemics**

**in Japan in the 1960s**

**Supplementary material**

# Supplementary Methods

**1. Model parameterizations**

For both independent and outbreak models, we employed four model versions: 1) the basic model, 2) a model with age-dependent force of infection (FOI), 3) a model with seroreversion, and 4) a model with both age-dependent FOI and seroreversion. The basic model followed the original assumptions on how the FOI changes over time **(Table S1)**. In the models with age-dependent FOI, the FOI also varied across different age groups, modulated by an age-related exponential coefficient. A positive coefficient indicated that the FOI increased with age, whereas a negative coefficient indicated that the FOI decreased with age. The models with seroreversion allowed seropositive individuals to return to the seronegative state, governed by a specified seroreversion rate. Finally, the models that included both an age-dependent FOI and seroreversion, integrated both the effects of age-related variation in FOI and the possibility of waning seropositivity over time.

**2. Calculating the probability of infection for a given age class in a given year**

Generally, we computed the attack rate (i.e. proportion of the population who gets infected) for each age class and each year using the parameter estimates from the serocatalytic models (including FOI estimates from 1958 to 1973) and a simple cohort model with a time step of a year. We consider the population 1-10 years old, and start the simulations in 1957, assuming the population is fully seronegative at that time point.

In the following expressions, we assume that $y$ is the sampling year. An individual whose age is $a$ was born on year $y-a$. This individual was exposed to $a$ forces of infection $\lambda_{i,j}$ where $i$ and $j$ are respectively, the year and age of exposure. Specifically, the FOIs are denoted as $\lambda_{y-a+1,1},\lambda_{y-a+2,2},\ldots,\lambda_{y,a}$ , representing the values of the force of infection from age 1 to $a$. For models accounting for variation in infection risk across different age groups, the time- and age-specific force of infection is given by $\lambda_{i,j}=\lambda_{i}e^{\beta\left( j-1 \right)},$where $\beta$ is the age-related coefficient. We denote by $S_{y-a,j}^{-}$ and $S_{y-a,j}^{+}$ the probability that an individual born on year $y-a$ is seronegative and seropositive at age $j$, hence, $S_{y-a,j}^{-}=1-S_{y-a,j}^{+}$. We further assume that children are fully susceptible at age 0, therefore, $S_{y-a,0}^{+}=0.$

The attack rate (AR) for individuals born on year $y-a$ at age $j$ is given by $S_{y-a,j-1}^{-}(1-e^{-\lambda_{y-a+j,j}})$. In particular, an individual of age $a=1$ is seronegative on year $y-1$ $\left( S_{y-1,0}^{-}=1 \right).$ Finally, the overall attack rate at a given year is obtained by taking the weighted average of the attack rates over different ages.

**2.1 Basic model**

The basic model assumes the same FOI across age and no seroreversion. Let $\lambda_{y-a, j}$ denote the FOI experienced by an individual born in year $y-a$ when they are $j$ years old, that is, the FOI in year $y-a+j$. The FOI only varied across years, but was the same across age groups:

$$\lambda_{y-a,j}=\lambda_{y-a+j}.$$

Then, the probability that an individual remains seronegative at age $j-1$ is given by:

$$S_{y-a, j-1}^{-}=\prod_{i=1}^{j-1} e^{-\lambda_{y-a, i}}=e^{-\sum_{i=1}^{j-1} \lambda_{y-a, i}}.$$

The annual attack rate for individuals of age $j$ in year $y-a+j$ is defined as:

$${AR}_{y-a+j}=S_{y-a, j-1}^{-}\cdot(1-e^{-\lambda_{y-a+j}}).$$

Here, the first term represents the probability of being susceptible at the beginning of the year, and the second term represents the probability of infection during that year.

**2.2 Model with age-dependent FOI**

In this model, the FOI varies by age and is modeled as a function of an age-related coefficient $\beta$. Let ​$\lambda_{y-a+j}$ denote the baseline FOI in calendar year $y-a+j$, and let $\beta$ be the age-scaling coefficient. The age-adjusted FOI experienced by an individual born in year $y-a$ at age $j$ is defined as:

$$\lambda_{y-a,j}=\lambda_{y-a+j}{.e}^{\beta(j-1)}.$$

Then, the probability that the individual remains seronegative at age $j-1$ is given by:

$$S_{y-a, j-1}^{-}=\prod_{i=1}^{j-1} e^{-\lambda_{y-a, i}}=e^{-\sum_{i=1}^{j-1} \lambda_{y-a, i}}.$$

The annual attack rate for individuals of age $j$ in year $y-a+j$ is defined as:

$${AR}_{y-a, j}=S_{y-a, j-1}^{-}\cdot\left( 1-e^{-\lambda_{y-a+j,j}} \right).$$

Here, the first term represents the probability of being susceptible at the beginning of the year, and the second term represents the probability of infection during that year.

**2.3 Model with seroreversion**

This model includes seroreversion, allowing seropositive individuals to revert to the seronegative state at rate $\rho$. Because the FOI varies with time and we assume seroreversion, we cannot write the probability of infection at a given year in a closed-form formula[1]. Instead, we have to solve recursively the serocatalytic differential equations at each year, assuming a constant force of infection during this year and taking the solution of the past year as the initial condition for the equation.

The probability that an individual of age $j$ is seropositive is the solution $P\left( 1 \right)$ of the differential equation

$$\frac{dP}{dt}=\lambda_{y-a,j}\left( 1-P \right)-\rho P$$

$$P\left( 0 \right)=S_{y-a,j-1}^{+}.$$

The solution is

$$S_{y-a,j}^{+}=\frac{\lambda_{y-a,j}}{\lambda_{y-a,j}+\rho}+\left( S_{y-a,j-1}^{+}-\frac{\lambda_{y-a,j}}{\lambda_{y-a,j}+\rho} \right)e^{{-(\lambda}_{y-a,j}+\rho)}.$$

The corresponding probability of being seronegative at age $j$ is:

$$S_{y-a, j}^{-}=1-S_{y-a, j}^{+}.$$

The annual attack rate for individuals of age $j$ in year $y-a+j$ is given by:

$${AR}_{y-a+j}=S_{y-a, j-1}^{-}\cdot\left( 1-e^{-\lambda_{y-a+j}} \right).$$

Here, $\lambda_{y-a+j}$​ is the force of infection experienced by an individual born in year $y-a$ at age $j$ (which is the same as other age groups in the same year). However, we have used, $S_{y-a,j-1}^{-}$ , the probability of being seronegative at the end of age $j-1$ to calculate the attack rate when the child entered age $j$.

**2.4 Model with age-dependent FOI and seroreversion**

This model incorporates both age-dependent FOI and seroreversion, allowing individuals to return to the susceptible state at a rate $\rho$, while also accounting for variation in infection risk across different age groups through an age-related coefficient $\beta$.

According to section 2.3, when accounting for seroreversion at a rate $\rho$ , we have:

$$S_{y-a,j}^{+}=\frac{\lambda_{y-a,j}}{\lambda_{y-a,j}+\rho}+\left( S_{y-a,j-1}^{+}-\frac{\lambda_{y-a,j}}{\lambda_{y-a,j}+\rho} \right)e^{{-(\lambda}_{y-a,j}+\rho)}.$$

Additionally, when accounting for age-dependent FOI, we have:

$$\lambda_{y-a,j}=\lambda_{y-a+j}{.e}^{\beta(j-1)}.$$

The corresponding probability of being seronegative at age $j$ in year $y-a+j$ is:

$$S_{y-a, j}^{-}=1-S_{y-a, j}^{+}.$$

The annual attack rate for individuals of age $j$ in year $y-a+j$ is defined as:

$${AR}_{y-a,j}=S_{y-a, j-1}^{-}\cdot\left( 1-e^{-\lambda_{y-a+j, j}} \right).$$

Here, $\lambda_{y-a+j, j}$​ is the force of infection experienced by an individual born in year $y-a$ at age $j$. $S_{y-a,j-1}^{-}$ is the probability of being seronegative at the end of age $j-1$. The annual attack rate is computed based on the probability of still being seronegative before the year and the FOI during the year, but the FOI also varied across different age groups.

**3. Computation and uncertainty**

We calculate age-specific attack rates over time for 1,000 randomly sampled draws from the posterior estimates of the models and report the median and the 95% credible intervals, computed as 2.5% and 97.5% quantiles.

# Table S1: Summary of model assumptions, parameters and prior distributions. For detailed descriptions of the models, see Hozé et al. [1]

|  | **Independent model** | **Outbreak model** |
| --- | --- | --- |
| Assumption | In the independent model, the FOI $\lambda$ is allowed to change every year. | The outbreak model imposes that there have been $K$ epidemics in the past, where $K$ is fixed. When $K$ = 1, the model referred to as the “one-outbreak model”; When $K$ = 2, the model referred to as the “two-outbreak model”. |
| Formula | We use year as the time unit. The probability that an individual of age $a$ is seropositive, $P(a)$, is given by the cumulative FOI of the individual from age $1$to $a$:  $P(a)=1-exp(-\sum_{i=1}^{a} \lambda_{i})$ | The FOI at year $i$ is given as a sum of $K$ Gaussians. Each Gaussian is centered on $T_{k}$, the peak epidemic time of the focal outbreak:  $\lambda_{i}=\sum_{k=1}^{K} \bar{\alpha}_{k}exp(-{(i-T_{k})}^{2})$  $\bar{\alpha}_{k}= \alpha_{k}\frac{1}{\sum_{k=1}^{K} exp(-{(i-T_{k})}^{2})}$ |
| Prior distributions | $\lambda$ : Normal (0.01, 1),  $\rho$: Normal (1, 1) | $\bar{\alpha}_{k}$ : Normal (0.2, 0.2)  $T_{k}$: Normal (20, 10)  $\rho$: Normal (1, 1) |

# Table S2: Model list and description.

| **Model name** | **Model description** |
| --- | --- |
| Model 1 | Independent model |
| Model 2 | Independent model with age-dependent FOI |
| Model 3 | Independent model with seroreversion |
| Model 4 | Independent model with age-dependent FOI and seroreversion |
| Model 5 | One-outbreak model |
| Model 6 | One-outbreak model with age-dependent FOI |
| Model 7 | One-outbreak model with seroreversion |
| Model 8 | One-outbreak model with age-dependent FOI and seroreversion |
| Model 9 | Two-outbreak model |
| Model 10 | Two -outbreak model with age-dependent FOI |
| Model 11 | Two -outbreak model with seroreversion |
| Model 12 | Two -outbreak model with age-dependent FOI and seroreversion |

# Table S3: Model comparison using the LOO and DIC criterion.

| **Model name** | **Model description** | ELPD DIFF | SE DIFF | DIC |
| --- | --- | --- | --- | --- |
| **Model 2** | **Independent model with age-dependent FOI** | **0** | **0** | **856.53** |
| **Model 3** | **Independent model model with seroreversion** | **-0.75** | **1.18** | **857.27** |
| **Model 4** | **Independent model with age-dependent FOI and seroreversion** | **-2.89** | **1.18** | **861.57** |
| **Model 11** | **Two-outbreak model with seroreversion** | **-4.41** | **5.22** | **866.47** |
| **Model 10** | **Two-outbreak model with age-dependent FOI** | **-6.36** | **6.21** | **870.13** |
| Model 1 | Independent model | -6.84 | 5.8 | 870.36 |
| Model 12 | Two-outbreak model with age-dependent FOI and seroreversion | -7.55 | 5.54 | 872.66 |
| Model 9 | Two-outbreak model | -14.93 | 7 | 886.58 |
| Model 5 | One-outbreak model | -64.89 | 14.86 | 986.8 |
| Model 7 | One-outbreak model with seroreversion | -78.31 | 20.45 | 1014.1 |
| Model 6 | One-outbreak model with age-dependent FOI | -78.82 | 19.14 | 1014.74 |
| Model 8 | One-outbreak model with age-dependent FOI and seroreversion | -81.56 | 20.52 | 1020.5 |

Abbreviation: ELPD_DIFF: Expected Log Pointwise Predictive Density Difference; SE_DIFF: Standard Error of the Difference; DIC: Deviance Information Criterion. The models highlighted in bold were the primary focus of our analysis.

# Table S4: Summary of the posterior estimates of the parameters.

| Parameters | | Independent model with age-dependent FOI (median, 95% CrI)* | Independent model with seroreversion  (median, 95% CrI) | Two-outbreak model with age-dependent FOI (median, 95% CrI)* | Two-outbreak model with seroreversion (median, 95% CrI) |
| --- | --- | --- | --- | --- | --- |
| FOI | 1958 | 0.01 (0.00-0.09) | 0.01 (0.00-0.09) | 0.00 (0.00-0.00) | 0.00 (0.00-0.00) |
|  | 1959 | 0.01 (0.00-0.11) | 0.01 (0.00-0.12) | 0.00 (0.00-0.01) | 0.00 (0.00-0.00) |
|  | 1960 | 0.02 (0.00-0.23) | 0.02 (0.00-0.26) | 0.01 (0.00-0.07) | 0.01 (0.00-0.07) |
|  | 1961 | 0.08 (0.00-0.35) | 0.12 (0.00-0.41) | 0.07 (0.01-0.15) | 0.07 (0.00-0.18) |
|  | 1962 | 0.02 (0.00-0.16) | 0.03 (0.00-0.22) | 0.09 (0.03-0.14) | 0.11 (0.03-0.17) |
|  | 1963 | 0.01 (0.00-0.06) | 0.01 (0.00-0.08) | 0.02 (0.00-0.09) | 0.03 (0.00-0.12) |
|  | 1964 | 0.01 (0.00-0.06) | 0.02 (0.00-0.07) | 0.00 (0.00-0.01) | 0.00 (0.00-0.04) |
|  | 1965 | 0.01 (0.00-0.03) | 0.01 (0.00-0.03) | 0.00 (0.00-0.00) | 0.00 (0.00-0.00) |
|  | 1966 | 0.01 (0.00-0.03) | 0.01 (0.00-0.03) | 0.00 (0.00-0.00) | 0.00 (0.00-0.00) |
|  | 1967 | 0.01 (0.00-0.04) | 0.01 (0.00-0.05) | 0.01 (0.00-0.01) | 0.01 (0.01-0.02) |
|  | 1968 | 0.12 (0.04-0.23) | 0.17 (0.08-0.27) | 0.10 (0.06-0.15) | 0.15 (0.10-0.21) |
|  | 1969 | 0.26 (0.16-0.41) | 0.29 (0.20-0.40) | 0.20 (0.16-0.26) | 0.26 (0.22-0.31) |
|  | 1970 | 0.02 (0.00-0.06) | 0.03 (0.01-0.08) | 0.06 (0.04-0.10) | 0.06 (0.04-0.10) |
|  | 1971 | 0.03 (0.01-0.08) | 0.04 (0.01-0.10) | 0.00 (0.00-0.01) | 0.00 (0.00-0.00) |
|  | 1972 | 0.01 (0.00-0.04) | 0.01 (0.00-0.04) | 0.00 (0.00-0.00) | 0.00 (0.00-0.00) |
|  | 1973 | 0.01 (0.00-0.03) | 0.01 (0.00-0.04) | 0.00 (0.00-0.00) | 0.00 (0.00-0.00) |
| Age-dependent FOI coefficient (age-risk) | | 0.01 (-0.05-0.08) | - | 0.04 (0-0.09) | - |
| Seroreversion rate | | - | 0.03 (0.01-0.08) | - | 0.02 (0.01-0.06) |
| Outbreak attack rate | | 36.9% (24.8-50.3%) for the later outbreak | 41.9% (36.4-47.9%) for the later outbreak | 19.8% (0.1-27.1%) for the earlier outbreak  34.9% (27.5-44.0%) for the lateroutbreak | 21.8% (0.1-29.7%) for the earlier outbreak  37.8% (33.3-43.5%) for the later outbreak |

* The FOI estimates were derived for 1-year-old children as the reference group. For other age groups, FOI was calculated by multiplying the reference FOI by *exp(age-risk× (j − 1))*, where j denotes the age of the group being estimated.

# Figure S1: Individual trajectories of the FOI estimations

Coloured lines and ribbons are the mean and 95% credible intervals (CrIs) estimated with the serocatalytic models. Grey lines are randomly sampled individual trajectories from the posterior distribution (n=20).


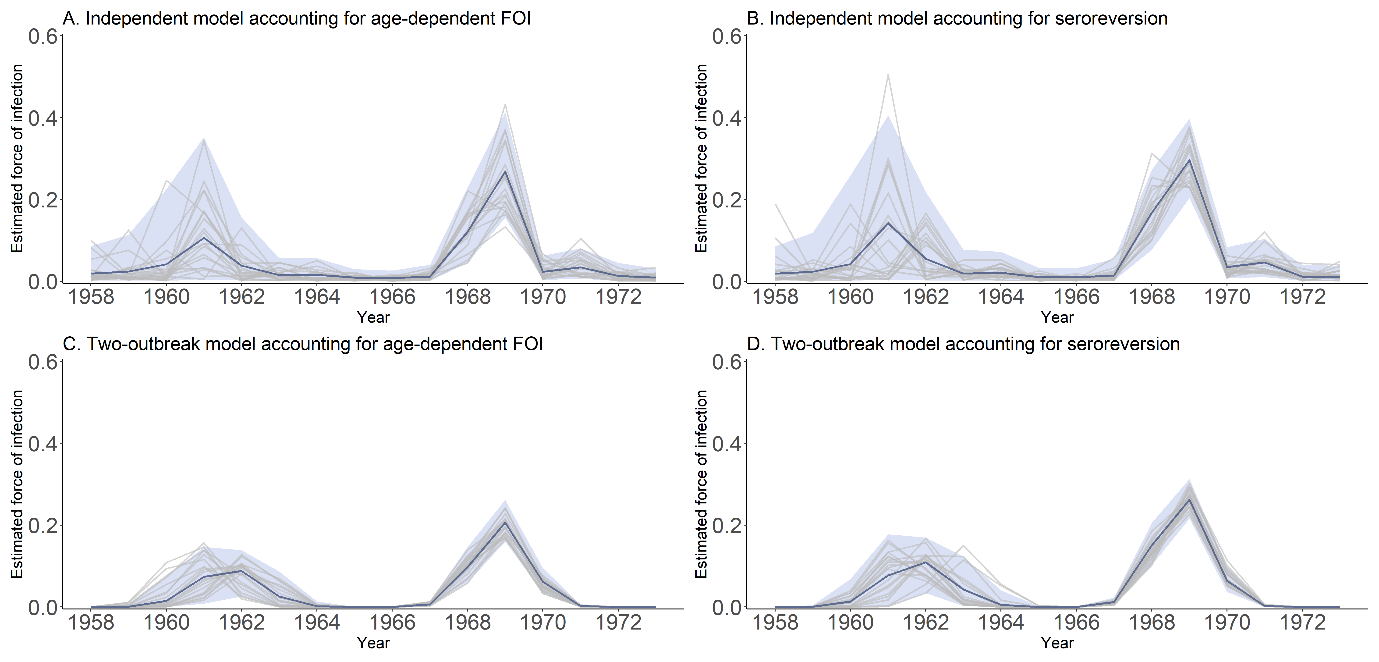


# Figure S2: Estimated fold change of the FOI with age for models accounting for age-dependent FOI (Model 2 and Model 10)


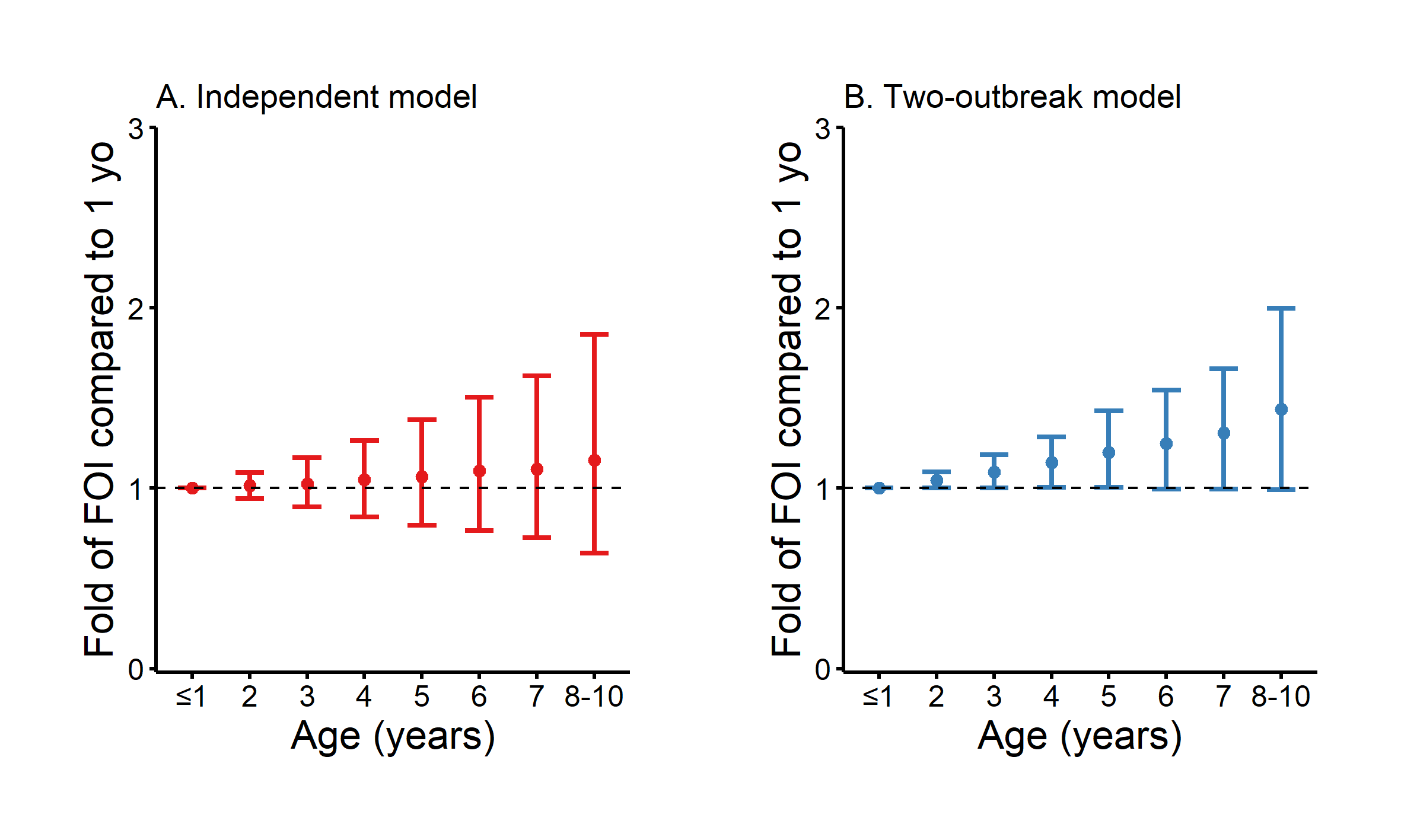


# Figure S3: Estimated median FOI by age for models accounting for age-dependent FOI (Model 2 and Model 10).


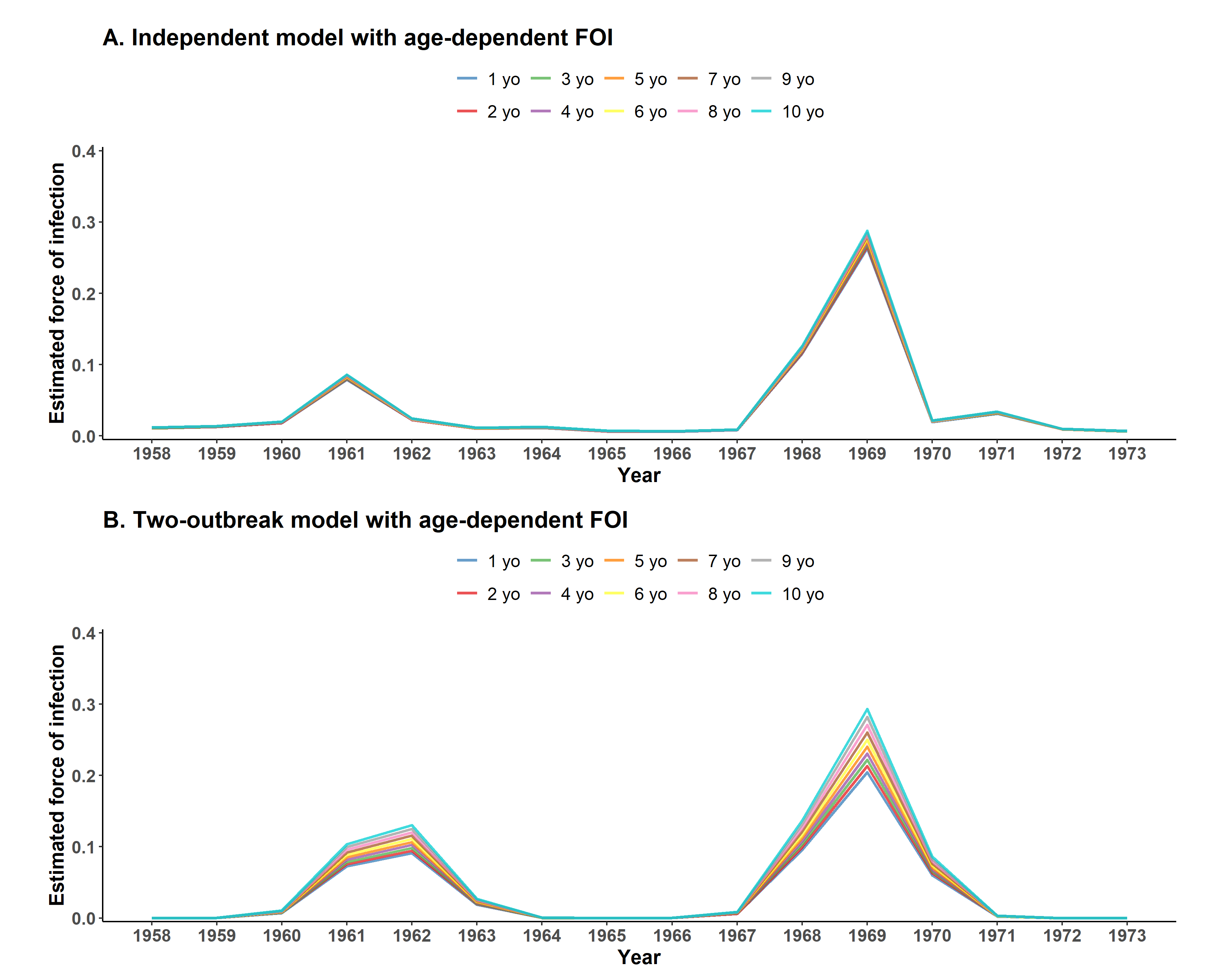


# Figure S4: Traceplot of each parameter for the independent model with age-dependent FOI.


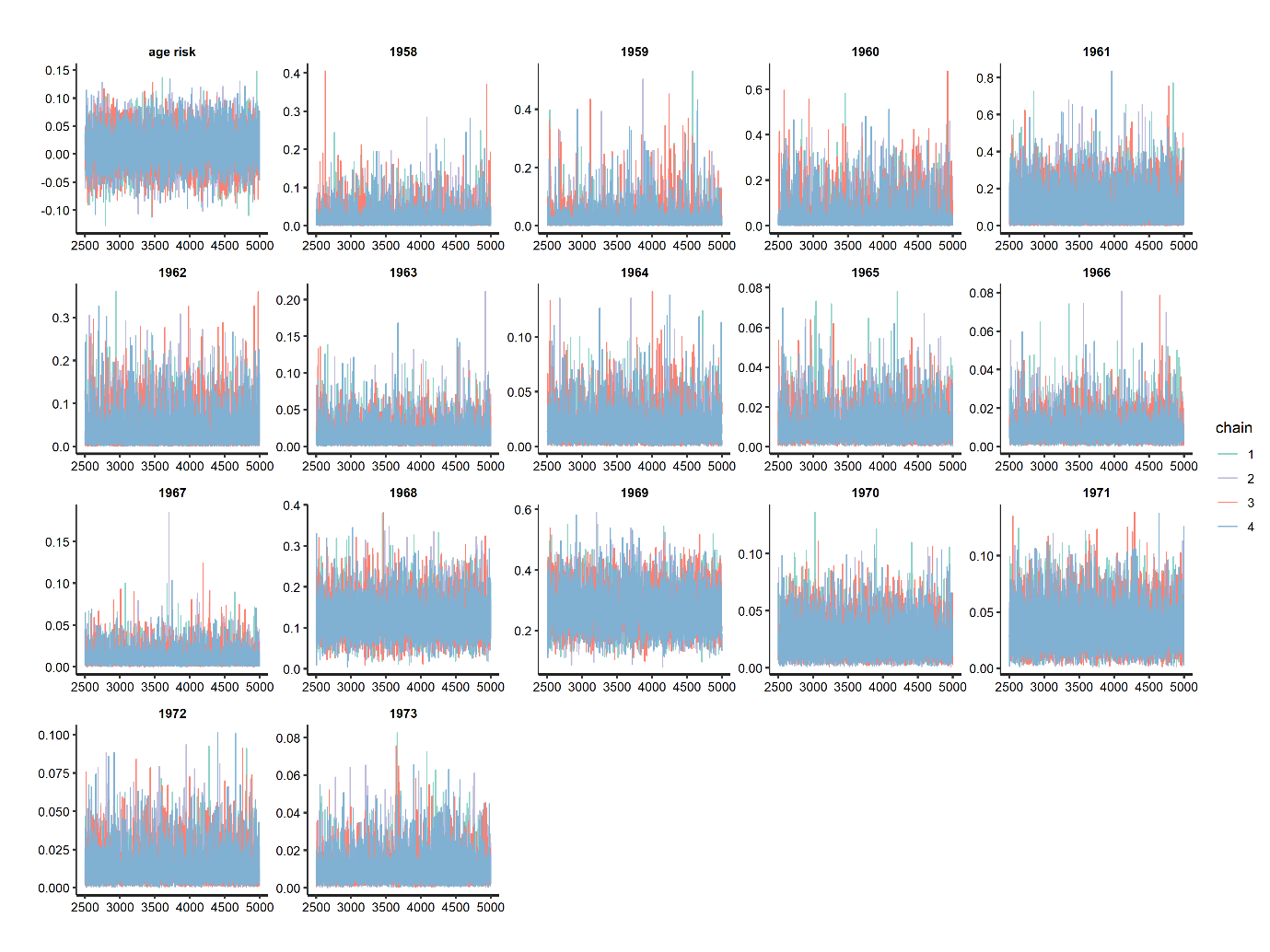


# Figure S5: Traceplot of each parameter for the independent model with seroreversion.


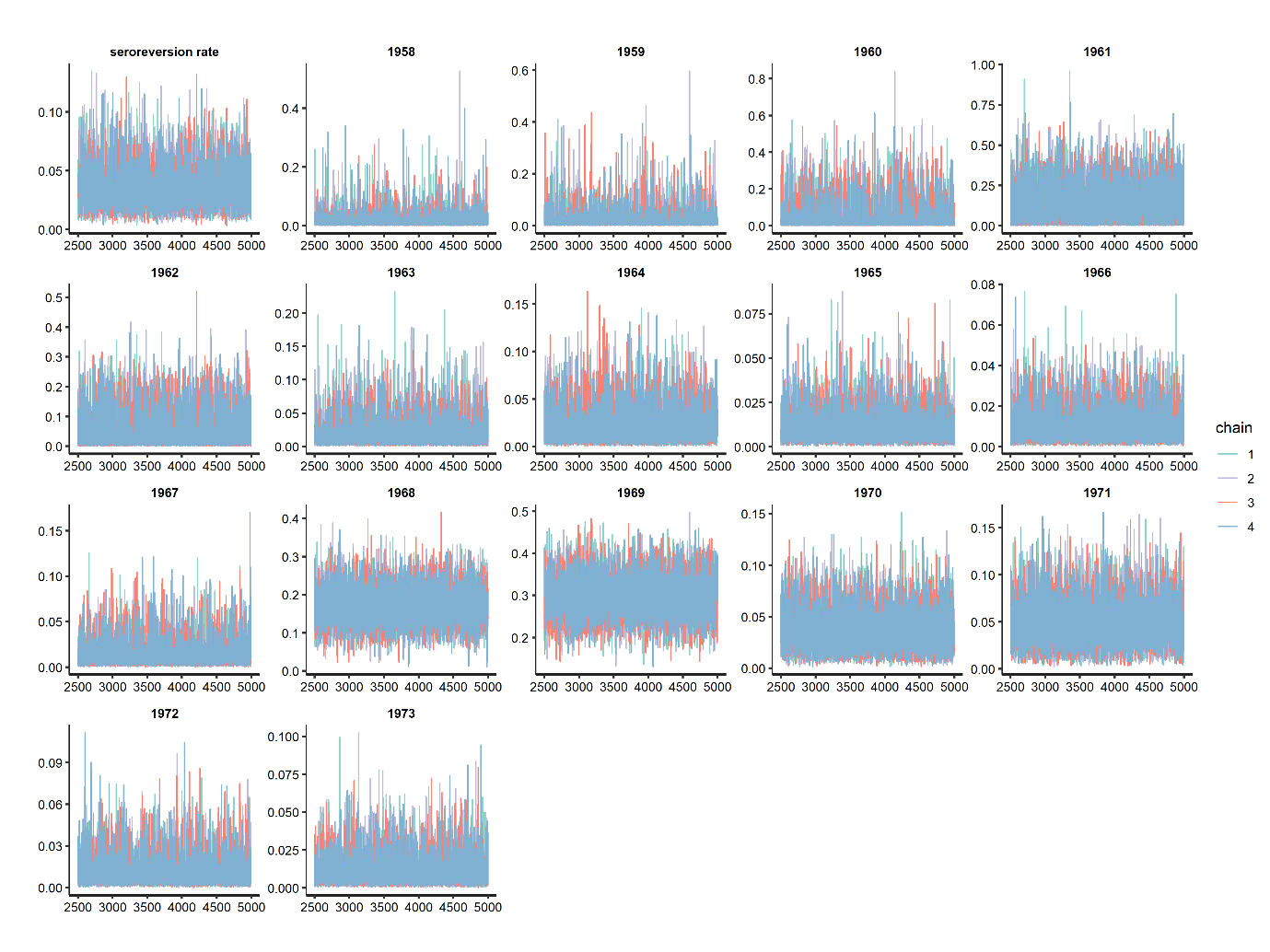


# Figure S6: Traceplot of each parameter for the two-outbreak model with age-dependent FOI.

The year of outbreak refers to the number of years prior to the final sampling year (1973) in our study. Alpha refers to a weighting factor used to represent the outbreak attack rate (Table S1).


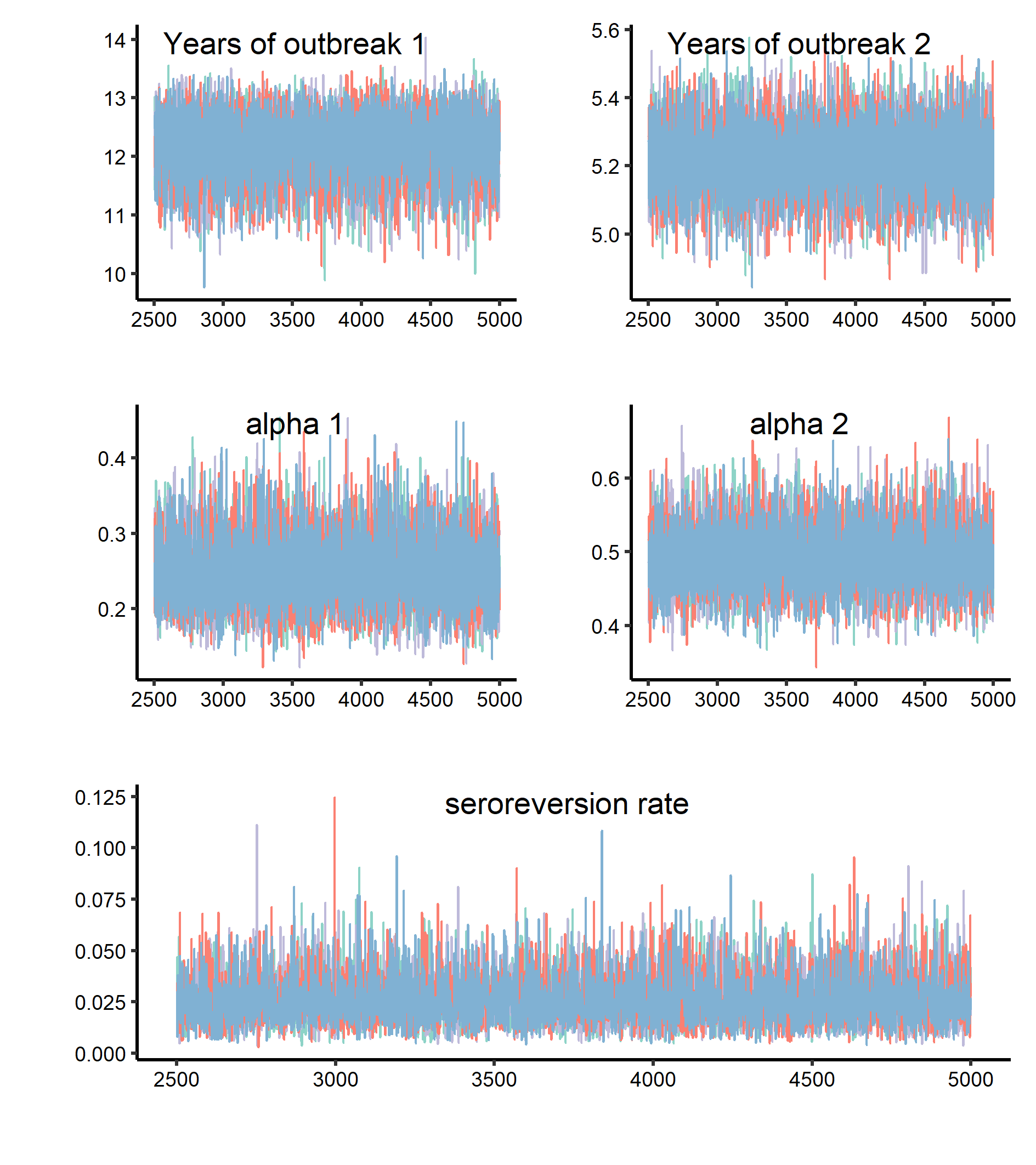


# Figure S7: Traceplot of each parameter for the independent models with seroreversion.

The year of outbreak refers to the number of years prior to the final sampling year (1973) in our study. Alpha refers to a weighting factor used to represent the outbreak attack rate (Table S1).


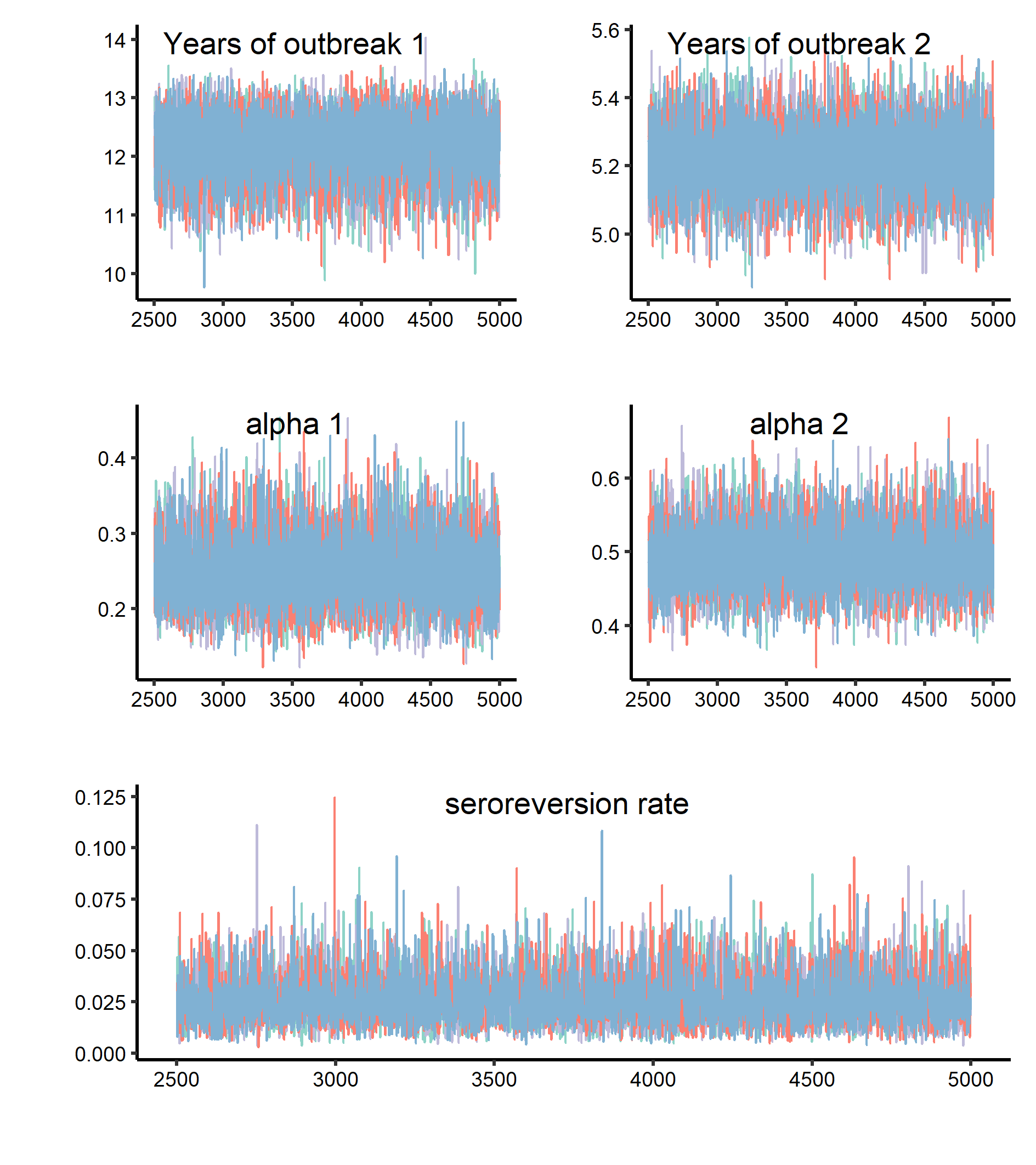


# Figure S8: Posterior density plots for each parameter for the independent model with age-dependent FOI.


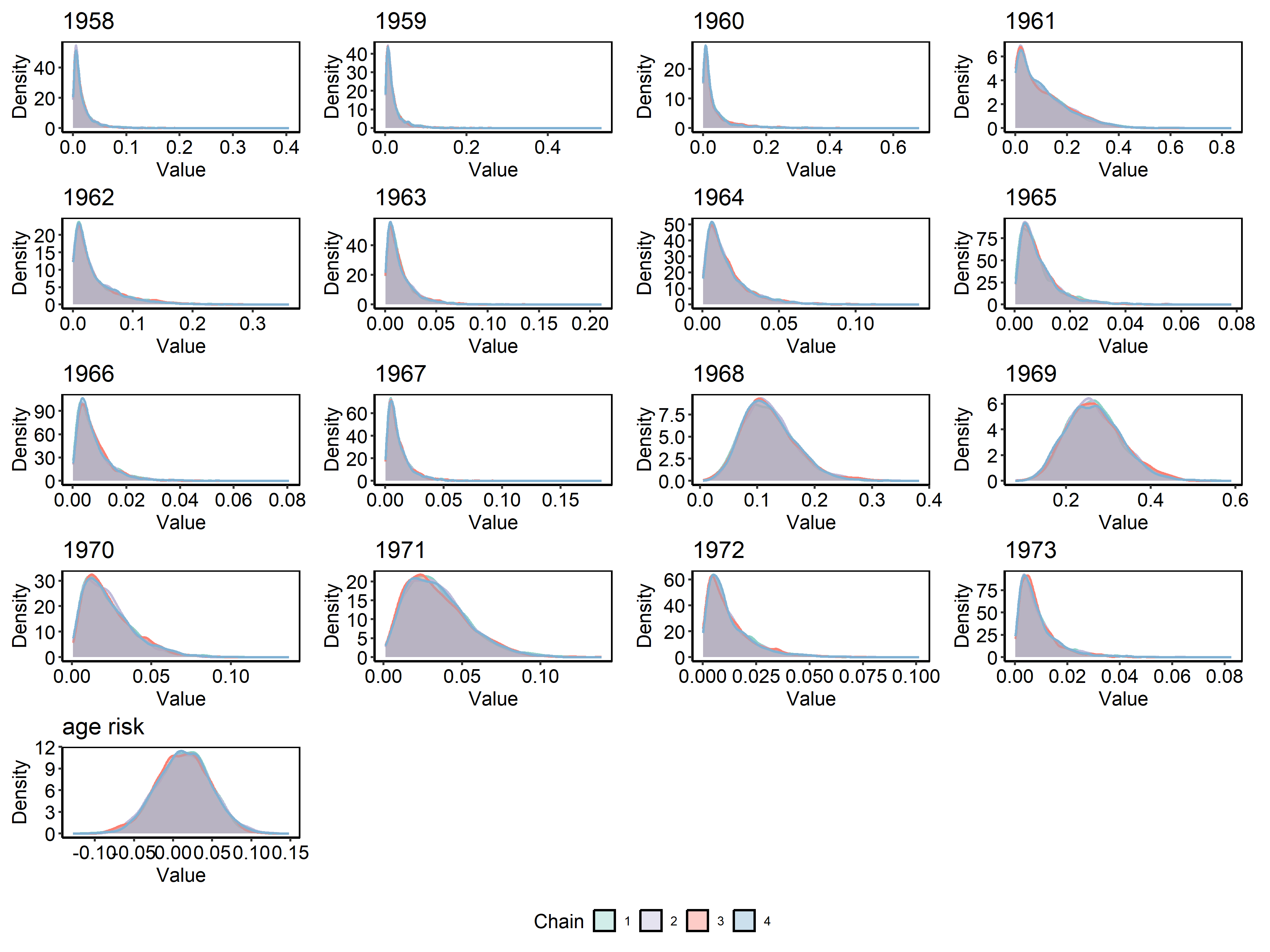


# Figure S9: Posterior density plots for each parameter for the independent model with seroreversion.


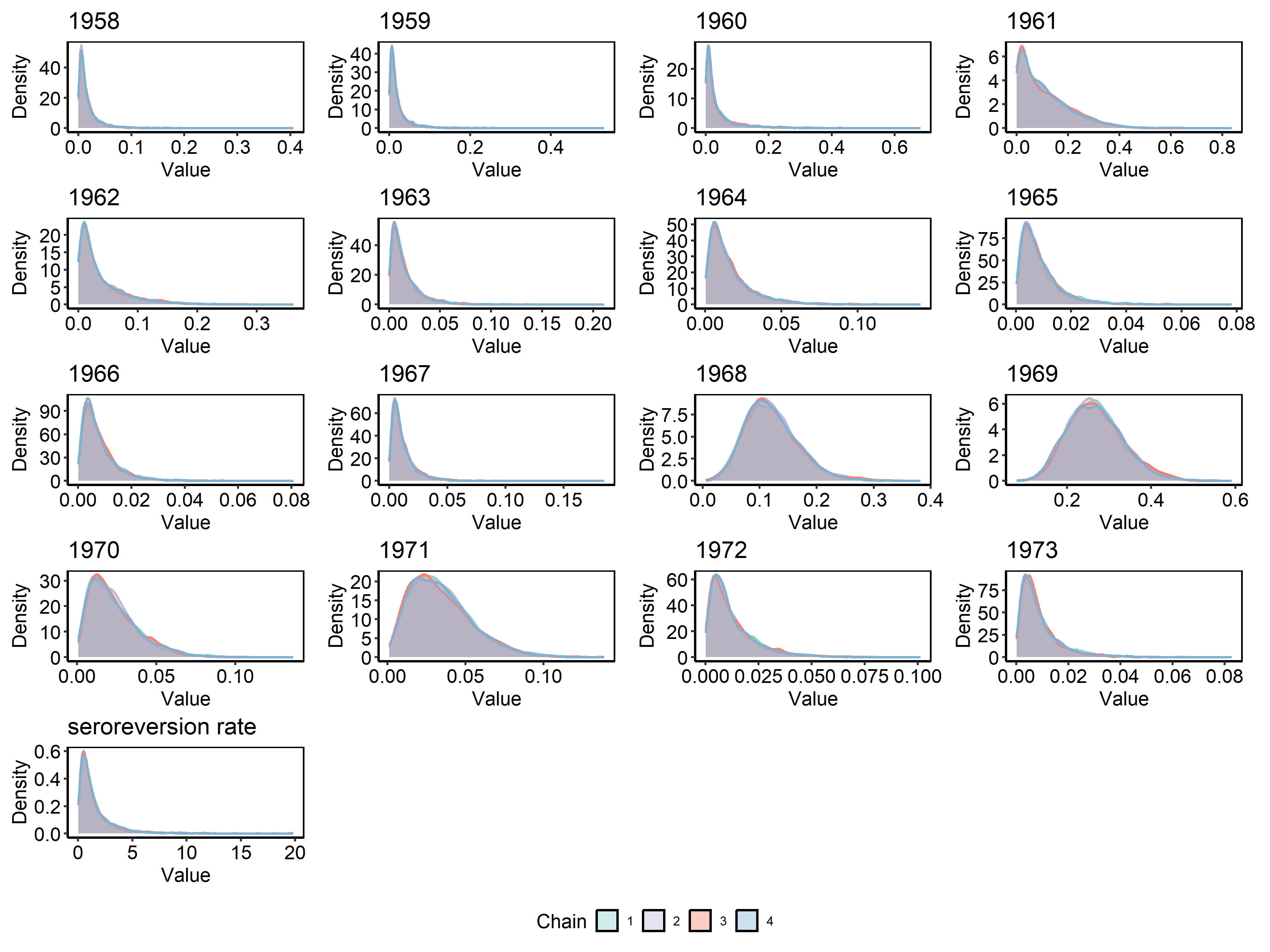


# Figure S10: Posterior density plots for each parameter for the two-outbreak model with age-dependent FOI.

The year of outbreak refers to the number of years prior to the final sampling year (1973) in our study. Alpha refers to a weighting factor used to represent the outbreak attack rate (Table S1).


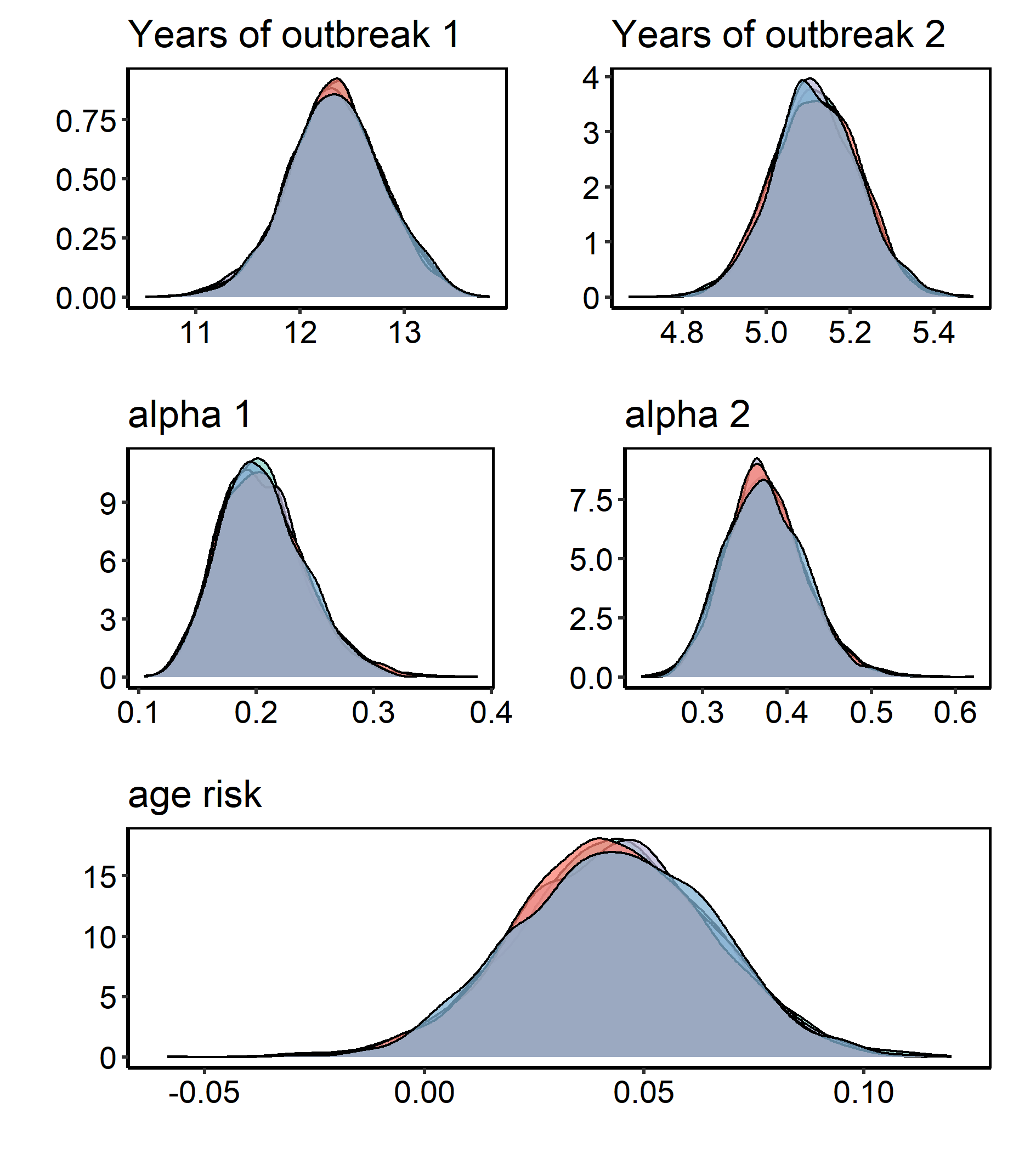


# Figure S11: Posterior density plots for each parameter for the two-outbreak model with seroreversion.

The year of outbreak refers to the number of years prior to the final sampling year (1973) in our study. Alpha refers to a weighting factor used to represent the outbreak attack rate (Table S1).


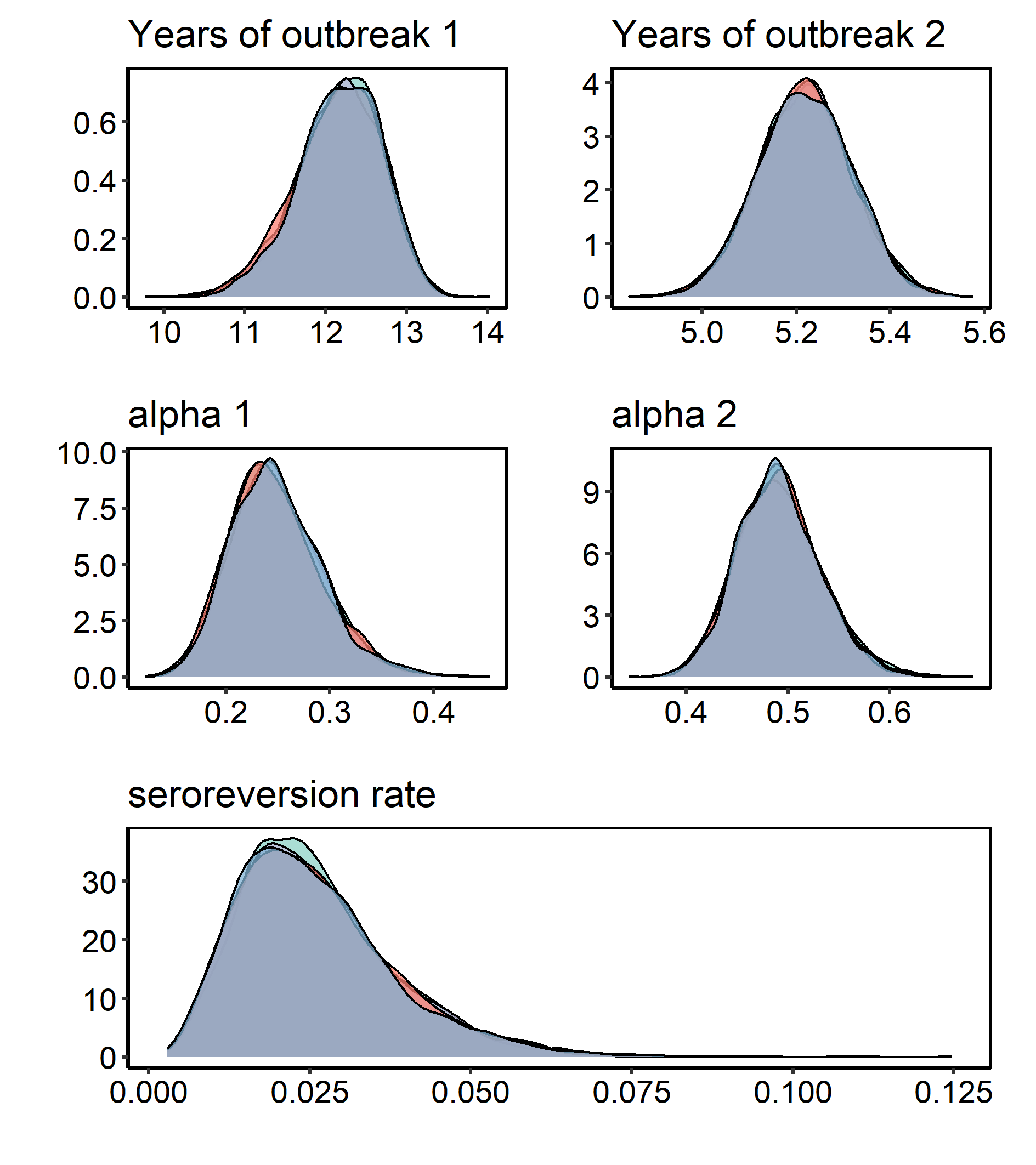


# References

1. Hozé N, Pons-Salort M, Metcalf CJE, White M, Salje H, Cauchemez S. RSero: A user-friendly R package to reconstruct pathogen circulation history from seroprevalence studies. PLOS Computational Biology **2025**; 21:e1012777.
